# Enhanced activation of dendritic cells by autologous apoptotic microvesicles in MRL/lpr mice

**DOI:** 10.1186/s13075-015-0617-2

**Published:** 2015-04-16

**Authors:** Jürgen Dieker, Luuk Hilbrands, Astrid Thielen, Henry Dijkman, Jo H Berden, Johan van der Vlag

**Affiliations:** Department of Nephrology, Radboud University Medical Centre, Nijmegen, The Netherlands; Department of Pathology, Radboud University Medical Centre, Nijmegen, The Netherlands; Department of Nephrology, Radboud Institute of Molecular Life Sciences, Radboud University Medical Centre, Geert Grooteplein 10, Nijmegen, 6525 GA The Netherlands

## Abstract

**Introduction:**

Systemic lupus erythematosus is associated with a persistent circulation of modified autoantigen-containing apoptotic debris that might be capable of breaking tolerance. We aimed to evaluate apoptotic microvesicles obtained from lupus or control mice for the presence of apoptosis-associated chromatin modifications and for their capacity to stimulate dendritic cells (DC) from lupus and control mice.

**Method:**

Apoptotic microvesicles were *in vitro* generated from splenocytes, and *ex vivo* isolated from plasma of both MRL/lpr lupus mice and normal BALB/c mice. Microvesicles were analyzed using flow cytometry. Bone marrow-derived (BM)-DC cultured from MRL/lpr or BALB/c mice were incubated with microvesicles and CD40 expression and cytokine production were determined as measure of activation.

**Results:**

Microvesicles derived from apoptotic splenocytes or plasma of MRL/lpr mice contained more modified chromatin compared to microvesicles of BALB/c mice, and showed enhanced activation of DC, either from MRL/lpr or BALB/c mice, and consecutively an enhanced DC-mediated activation of splenocytes. The content of apoptosis-modified chromatin in microvesicles of apoptotic splenocytes correlated with their potency to induce interleukin-6 (IL-6) production by DC. Microvesicle-activated MRL/lpr DC showed a significant higher production of IL-6 and tumor growth factor-β (TGF-β) compared to BALB/c DC, and were more potent in the activation of splenocytes.

**Conclusion:**

Apoptotic microvesicles from MRL/lpr mice are more potent activators of DC, and DC from MRL/lpr mice appear relatively more sensitive to activation by apoptotic microvesicles. Our findings indicate that aberrations at the level of apoptotic microvesicles and possibly DC contribute to the autoimmune response against chromatin in MRL/lpr mice.

## Introduction

Systemic lupus erythematosus (SLE) is a systemic autoimmune disease characterized by high levels of autoantibodies against chromatin [1]. Disturbances in apoptosis and the removal of apoptotic cells, leading to the persistent presence of apoptotic debris in the circulation, have been associated with the development of SLE in patients and in mouse models [2,3]. Apoptosis involves the translocation of chromatin from the nucleus into MVs (MVs) at the cell surface. In case of prolonged circulation of apoptotic cells, the MVs can be released and expose their contents to the immune system [4,5]. Chromatin is present in the circulation of SLE patients and lupus mice [6-8]. Subsequent formation and deposition of chromatin-anti-chromatin complexes, primarily in the skin and kidneys, elicits devastating local inflammation [9,10].

By recognizing dying cells as a danger sign, the immune system plays an important role in monitoring the health status of an organism [11]. We have previously demonstrated that apoptotic MVs can activate dendritic cells (DC) by inducing an increased expression of co-stimulatory molecules CD40 and CD86, and an increased production of pro-inflammatory cytokines IL-6 and tumor necrosis factor-α (TNF-α) [12,13]. The exposure of DC to different types of apoptotic cells may guide the immune response towards tolerance or autoimmunity, and the corresponding type of T cell polarization (Th1, Th2, Th17 or Treg) [14].

In the circulation, apoptotic MVs are part of a larger population of MVs, which are defined as small particles with a diameter of 100 to 1000 nm formed by blebbing of the plasma membrane [15]. MVs are released by platelets, red blood cells, endothelial cells, and T and B cells. MVs play a role in coagulation, secretion of cytokines, and intercellular communication. Changes in the concentration or composition of circulating MVs have been associated with rheumatoid arthritis, systemic sclerosis, polymyositis/dermatomyositis, and SLE [16-19]. Studies on the contents and role of circulating (apoptotic) MVs in lupus mice and patients are scarce and largely limited to *in vitro*-generated apoptotic MVs, which indeed contain lupus autoantigens, such as chromatin, and are recognized by lupus-derived autoantibodies [20-23].

Recognition of apoptotic chromatin by the immune system could be facilitated by the generation of apoptosis-associated post-translational modifications [24]. Previously, we showed the relevance of apoptosis-associated chromatin modifications. We identified histone H4-K8,12,16 ac, H2B-K12ac, H3-K27me3 and conformational acetylated chromatin epitopes as targets of antibodies present in the serum of lupus mice and SLE patients [25-28]. The identified chromatin modifications were enriched during apoptosis and were present in circulating chromatin in SLE patients. In addition, hyperacetylated nucleosomes, in contrast to normally acetylated nucleosomes, were superior in maturation of DC, whereas a peptide containing H4-K8ac,12 ac,16 ac led to acceleration of disease onset and aggravation of disease symptoms in pre-diseased lupus mice [25].

In the present study, we evaluated whether the impact of apoptotic MVs on dendritic cells was different in lupus mice compared to control mice. We compared both apoptotic MVs, generated either *in vitro* or *in vivo*, and DC from lupus and normal mice.

## Materials and methods

### Cell culture

Murine 32D clone 3 (32Dcl3) cells were cultured in complete medium as described [13]. BALB/c, CBA and MRL/lpr mice were purchased (Harlan, Blackthorn, UK), and maintained under specific pathogen-free conditions and handled according to the guidelines of the local ethics committee of the Radboud University in Nijmegen. The mice used in this study were 8 to 10 weeks old and experienced no active lupus disease, which was determined by measuring anti-dsDNA antibodies in ELISA, as previously described (25), and by measuring proteinuria using Albustix (Siemens Healthcare Diagnostics, Munich, Germany). Bone marrow-derived DC were obtained by culturing bone marrow from the respective mice as previously described [29,30]. Mixed lymphocyte reaction (MLR) of MV-activated DC and CBA splenocytes was performed as described earlier [13]. For proliferation assays, splenocytes were labeled with carboxyfluorescein succinimidyl ester (CFSE, Molecular Probes, Life Technologies, Paisley, UK) according to the manufacturer’s protocol.

### Induction of apoptosis and isolation of apoptotic microvesicles

Apoptosis was induced by incubating cells for 16 hours with 10 μM 4-nitroquinoline 1-oxide (4-NQO; Sigma-Aldrich, Zwijndrecht, The Netherlands). Apoptosis was measured using fluorescein isothiocyanate (FITC)-conjugated annexin V and propidium iodide (PI) according to the manufacturers’ protocol (BioVision, Palo Alto, USA). MVs were isolated from apoptotic 32Dcl3 cells and splenocytes as described previously [13]. Shortly, cells were removed by centrifugation for 10 minutes at 1,550 g at 4°C, and apoptotic MVs were isolated from the resulting supernatant by centrifugation for 50 minutes at 15,700 g (Sorvall RC-6 Centrifuge, SS34 rotor; Thermoscientific, Waltham, USA) at 4°C. In addition, MVs were isolated from frozen EDTA-plasmas of 8- to 10-week-old BALB/c, CBA and MRL/lpr mice. For this, plasma samples were centrifuged twice for 5 minutes at 500 g at 4°C to remove residual cells, after which the supernatant was centrifuged for 20 minutes at 20.800 g at 4°C (Eppendorf 5417R Centrifuge; Eppendorf, Hamburg, Germany). For addition to DC, MVs were isolated according to the method described above, subsequently the MV pellet was resuspended in RPMI medium without FCS, and the protein concentration was determined using the bicinchoninic acid assay (Sigma-Aldrich). Hereafter, FCS was added to the MV preparation to achieve a final concentration of 10%. The effect of MVs on DC was determined by adding equal amounts in protein equivalents of MVs for 16 hours at day 8 of the DC culture. Plasma-derived MVs isolated from 25 μl plasma of MRL/lpr or BALB/c mice were reconstituted in 25 μl medium, adjusted to acquire equal amounts in protein equivalents, and 2 or 10 μl was added to DC in a 96-well plate containing 200 μl medium per well. Lipopolysaccharide (LPS) (1 μg/ml; tlrl-3pelps, InvivoGen, San Diego, USA) was used as a positive control.

### Sodium dodecyl sulfate-polyacrylamide gel electrophoresis (SDS-PAGE) and western blotting

Cell extracts were prepared by dissolving the pelleted MVs in one volume of PBS and adding two volumes of 3 × Laemmli buffer (62.5 mM Tris-HCl pH 6.8, 25% glycerol, 2% SDS, 0.01% bromophenol Blue, 5% β-mercaptoethanol). Samples were heated for 5 minutes at 95°C, resolved by SDS-PAGE (18%) and transferred to nitrocellulose as previously described [25].

### Confocal and electron microscopy

For immune fluorescence staining, cytospun cells were fixed with 2% paraformaldehyde, permeabilized with 0.3% Triton X-100 and incubated with the indicated mouse chromatin monoclonal antibodies (mAbs) followed by Alexa 488-conjugated goat anti-mouse Ig(H + L) (Life Technologies, Naarden, The Netherlands). Samples were visualized using a Leica SP5 confocal microscope (Leica, Heidelberg, Germany). For transmission electron microscopy (TEM), MVs were fixed with 2.5% glutaraldehyde, postfixed in cacodylate-buffered 1% OsO_4_ and incubated with 50% alcohol/2% uranyl acetate. Subsequently, samples were dehydrated and embedded in EPON 812. Ultrathin sections were cut on an ultratome (Leica Microsystems, Heidelberg, Germany) and samples were examined by electron microscopy (Jeol 1200 EX2; Jeol, Nieuw-Vennep, The Netherlands).

### Flow cytometry and enzyme-linked immunosorbent assay (ELISA)

MVs were washed using PBS with 0.1% BSA (PBA) and incubated with the indicated lupus-mouse derived mAbs or mouse IgG2A isotype (Sigma), followed by Alexa 488-conjugated goat anti-mouse Ig(H + L) (Life Technologies). MVs were also incubated with PE-conjugated anti-CD3 (clone 17A2), anti-CD19 (clone 1D3) or rat IgG2A isotype (clone eBR2A) (all BD Biosciences) or with FITC-conjugated annexin V according to the manufacturers’ protocol (BioVision). If indicated, FIX & PERM® (Life technologies) was used for fixation/permeabilization of the MVs according to the manufacturer’s protocol. The size of the MV population was estimated by flow cytometry using calibrated nanobeads (Spherotech, Lake Forest, IL, USA). The concentration of MVs was determined by adding a known concentration of calibrated beads (AccuCheck, Life Technologies) according to the manufacturer’s protocol. Splenocytes were washed with PBA and subsequently incubated with PE-conjugated anti-CD3 in combination with FITC-conjugated anti-CD4 (clone H129.19) or anti-CD8 (clone 53-6.7), or PE-conjugated anti-CD19 (all BD Biosciences). DC were stained with PE-conjugated anti-CD40 (clone FGK45.5; Miltenyi Biotec, Utrecht, The Netherlands) and Alexa 647-conjugated anti-CD11c (clone N418; Serotec, Oxford, UK). Samples were analyzed using a FACSCalibur flow cytometer (BD Biosciences, San Jose, CA, USA). Levels of TNF-α, IL-6, and transforming growth factor (TGF)-β in supernatants of DC cultures, or IFN-γ in supernatants from MLR cultures, were determined in ELISA according to the manufacturer’s protocol (eBioscience, San Diego, CA, USA). Relative production was calculated compared to the respective unstimulated DC, and expressed as fold-induction.

### Statistical analysis

Values are expressed as mean ± standard error of the mean (SEM), and significance was determined by Student’s *t*-test or Mann-Whitney *U*-test using GraphPad Prism (GraphPad Software, San Diego, CA, USA). *P*-values less than 0.05 were considered as statistically significant.

## Results

### The 32Dcl3-derived microvesicles contain apoptosis-associated chromatin modifications

**Figure 1:**
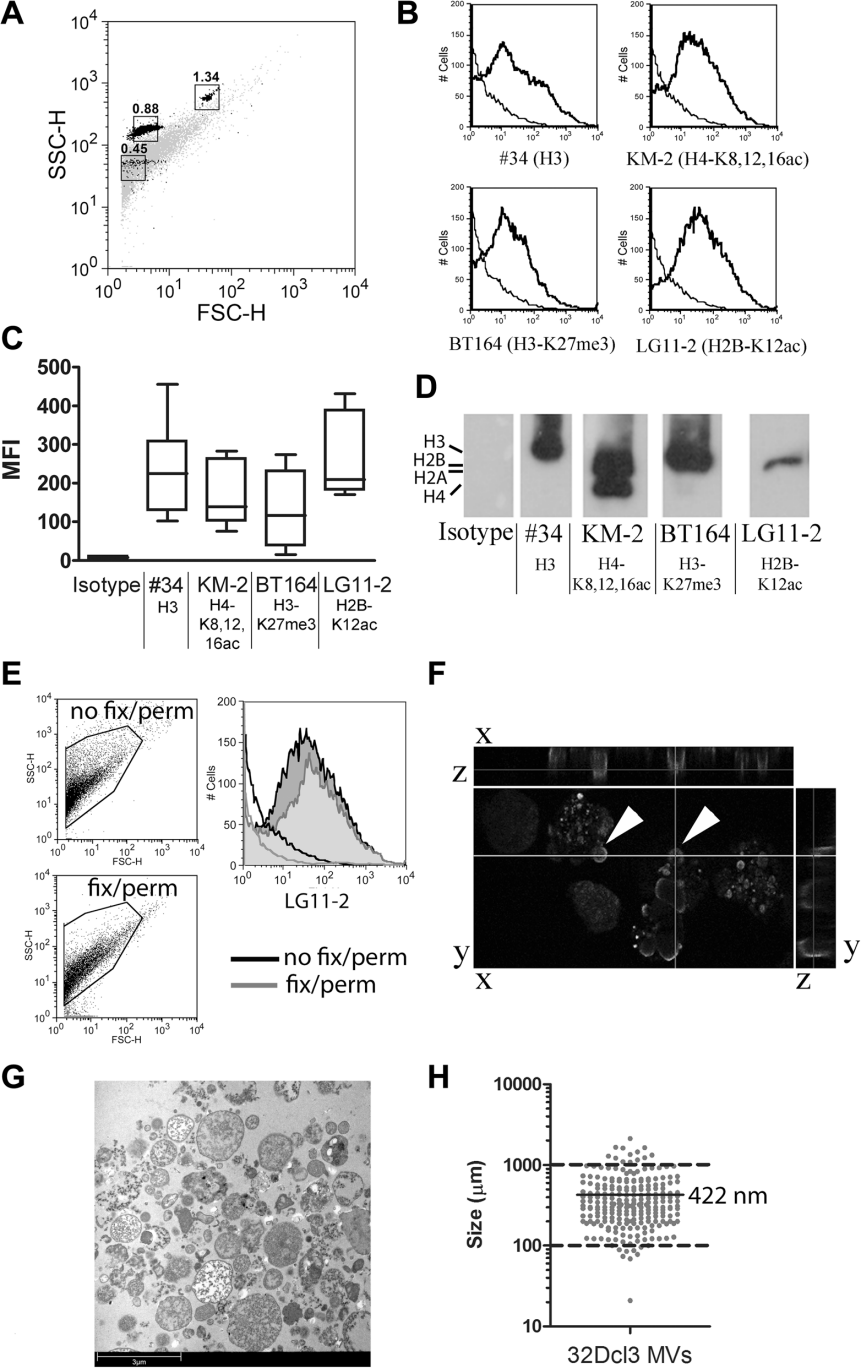
Apoptotic microvesicles derived from 32Dcl3 cells contain modified chromatin. **(A)** Forward scatter/side scatter (FSC/SSC) plot of microvesicles isolated from apoptotic 32Dcl3 cells, indicating the size of the microvesicle population (gray) according to calibrated nanobeads with the indicated size in μm (black). **(B)** 32Dcl3-derived apoptotic microvesicles were stained in flow cytometry for lupus mouse-derived mAbs #34, KM-2, BT164 and LG11-2. Histograms for a representative experiment are shown and the isotype control is indicated in each graph (fine line). **(C)** Mean fluorescence intensity (MFI) of for the indicated mAbs in multiple experiments (n = 10) showing the signal-range (25 to 75%) between different microvesicle preparations. **(D)** Extracts of 32Dcl3-derived apoptotic microvesicles were probed in western blot with the indicated lupus mouse-derived mAbs to confirm the presence of apoptosis-modified histones. The expected position of the respective histones is indicated on the left side. **(E)** 32Dcl3-derived apoptotic microvesicles were either directly stained (no fix/perm), or fixed and permeabilized before staining (fix/perm), and show no differences for the signal of LG11-2. FSC/SSC plots of the microvesicle population for both conditions are indicated on the left. On the right a representative histogram is shown for the isotype control (not filled) and mAb LG11-2 (filled) for microvesicles without (black line) or with fixation/permeabilization (gray line). **(F)** Confocal microscopy of 32Dcl3 cells stained with mAb KM-2. Apoptotic microvesicles still attached to early apoptotic 32Dcl3 cells are indicated (arrowheads). **(G)** Ultra-structural analysis of isolated 32Dcl3-derived apoptotic microvesicles by electron microscopy. Bar, 3 μm. **(H)** Median size of isolated microvesicles as determined by measuring the size in multiple electron microscopy pictures.

Apoptotic MVs from 4-NQO-treated 32Dcl3 granulocyte cells were stained with lupus mouse-derived mAbs #34 (anti-H3.1) [31], KM-2 (anti-H2A/H4-K8,12,16 ac) [25], BT164 (anti-H3-K27me3) [26], and LG11-2 (anti-H2B-K12ac) [27]. Note that we have previously associated the chromatin modifications recognized by KM-2, LG11-2 and BT164 with apoptosis and SLE [25-27]. The gated population of MVs (Figure 1A) stained positive for the tested mAbs (Figure 1B) with some degree of variability (Figure 1C). Western blot analysis (Figure 1D) confirmed the presence of apoptosis-modified histones in 32Dcl3-derived MVs. Permeabilization and fixation did not significantly alter the MV population based on the FSC/SSC plot and the staining by any of the mAbs (Figure 1E, LG11-2 is shown as a representative example). In confocal microscopy, the (modified) histones appeared primarily located on the outer rim of MVs that were still attached to the apoptotic cell (Figure 1F). Analysis of 32Dcl3-derived MVs by electron microscopy showed a large variety of MVs, of which a representative example is shown in Figure 1G. The median size of the isolated MVs, as observed in multiple electron microscopy pictures, was 422 nm, with only 3.4% of the MVs below 0.1 μm and 0.4% above 1 μm (Figure 1H). In summary, we detected apoptosis-associated chromatin modifications in 32Dcl3-derived apoptotic MVs that were located predominantly at the outer rim of MVs.

### MRL/lpr DC are relatively more sensitive to activation by 32Dcl3-derived than BALB/c DC

**Figure 2:**
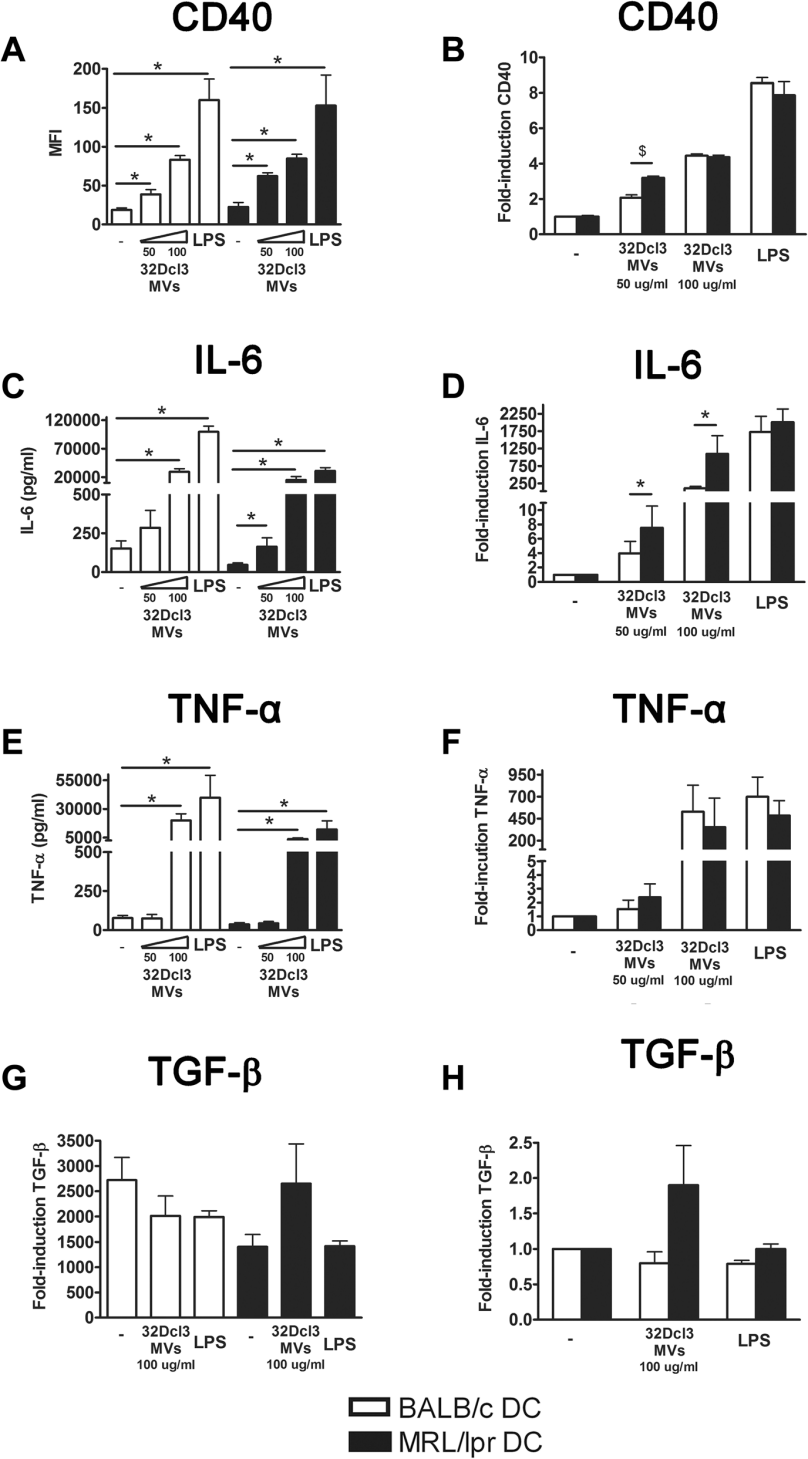
MRL/lpr dendritic cells (DC) are more prone to activation by 32Dcl3-derived apoptotic microvesicles, compared to BALB/c DC. CD40 expression **(A)**, and IL-6 **(C)**, TNF-α **(E)** and transforming growth factor (TGF)-β **(G)** levels produced by BALB/c and MRL/lpr DC were measured after stimulation with the indicated concentration of 32Dcl3-derived apoptotic microvesicles. The relative CD40 expression **(B)**, and IL-6 **(D)**, TNF-α **(F)** and TGF-β **(H)** levels of microvesicle- or lipopolysaccharide (LPS)-stimulated MRL/lpr DC were compared to BALB/c DC. *P <0.05 compared to unstimulated DC, ^$^ *P* <0.05 compared to DC from BALB/c. MFI, mean fluorescence intensity.

The response of cultured DC from lupus mice and normal mice to MVs from 4-NQO-treated 32Dcl3 cells revealed induction of CD40 that was significantly higher in MRL/lpr DC using 50 μg/ml MVs, while it was comparable with normal mice using 100 μg/ml MVs or LPS (Figure 2A and B). MRL/lpr DC that were unstimulated or stimulated with LPS, produced significantly lower IL-6 levels compared to BALB/c DC (Figure 2C). However, the induction of IL-6 by MVs was 2- to 3-fold higher in MRL/lpr DC compared with BALB/c DC (Figure 2D). TNF-α was also induced by addition of MVs, but with no significant differences between MRL/lpr and BALB/c DC (Figure 2E and F). MRL/lpr DC seem to produce lower basal levels of TGF-β compared with BALB/c DC (*P* = 0.10), while MVs tended to induce a higher TGF-β production in MRL/lpr DC (*P* = 0.14) (Figure 2G and H). In conclusion, with respect to CD40 upregulation and IL-6 production, DC from MRL/lpr mice appeared to be relatively more sensitive to stimulation by 32Dcl3-derived MVs than BALB/c DC.

### Splenocyte-derived apoptotic microvesicles from MRL/lpr mice expose more chromatin than those from BALB/c mice

**Figure 3:**
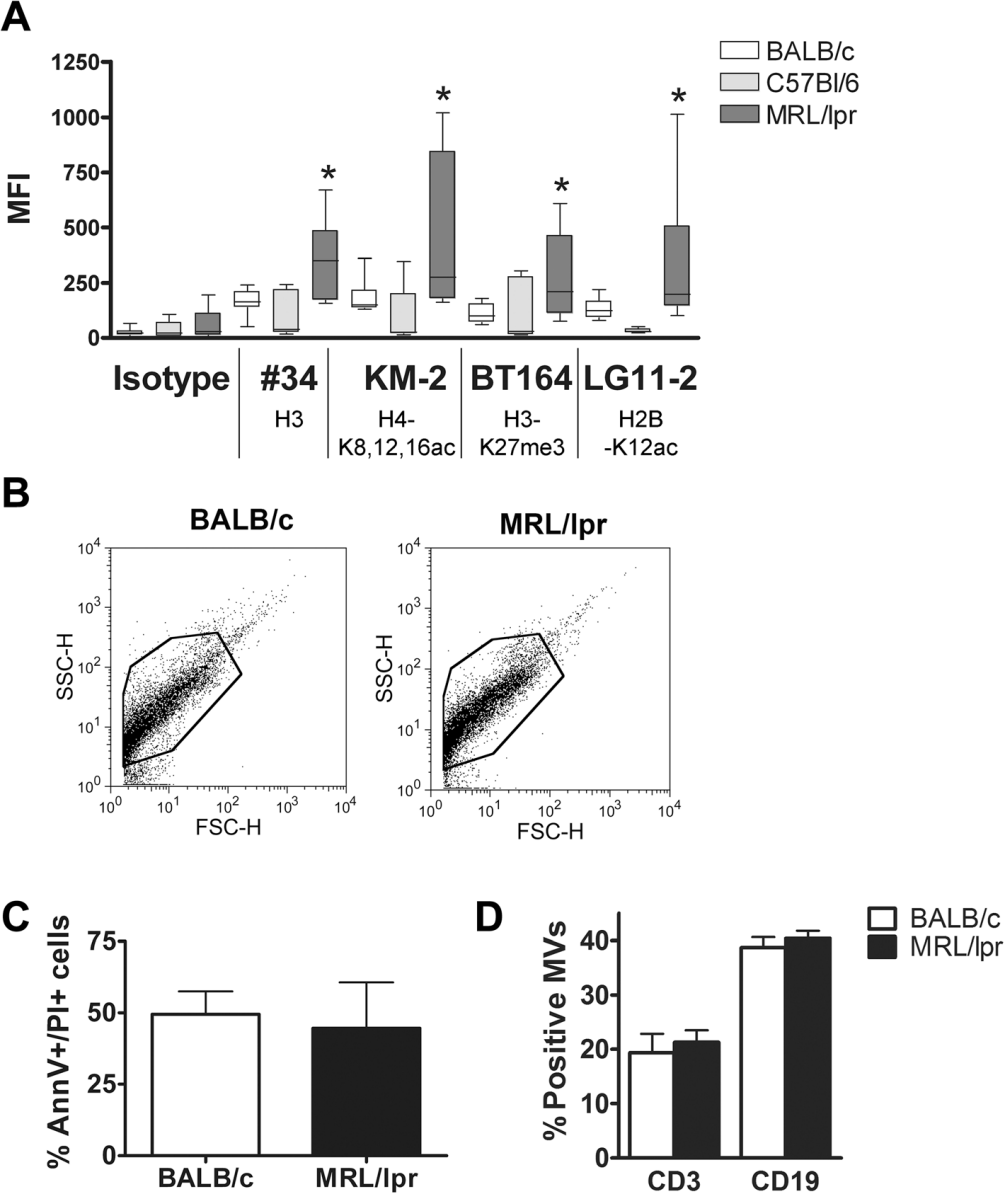
Splenocyte-derived apoptotic microvesicles from MRL/lpr mice contain more (modified) chromatin compared to BALB/c splenocyte-derived microvesicles. **(A)** Microvesicles from apoptotic splenocytes of 8- to 10-week-old MRL/lpr mice (n = 12) were stained for lupus-mouse derived mAbs #34, KM-2, BT164 and LG11-2, and compared to splenocytes from BALB/c (n = 11) and C57Bl/6 mice (n = 7). *P <0.05. **(B)** Representative example of a forward scatter/side scatter (FSC/SSC) plot for BALB/c and MRL/lpr splenocyte-derived apoptotic microvesicles demonstrating comparable microvesicle-populations. **(C)** 4-NQO-induced apoptosis for BALB/c and MRL/lpr splenocytes after 16 hours was determined by flow cytometry using annexin V-fluorescein isothiocyanate (FITC) and propium iodide (PI). **(D)** The percentage of CD3- and CD19-positive apoptotic microvesicles for splenocytes of MRL/lpr and BALB/c mice 16 hours after induction of apoptosis was measured by flow cytometry.

MRL/lpr (8- to 10-week-old) splenocyte-derived MVs demonstrated a higher mean fluorescence intensity (MFI) after staining with mAb #34 (against unmodified chromatin), and with KM-2, BT164 and LG11-2 (all against apoptosis-modified chromatin), when compared to MVs derived from BALB/c or C57BL/6 splenocytes (Figure 3A). We did not observe obvious differences in the MV population of MRL/lpr and BALB/c mice in forward scatter (FSC)/side scatter (SSC) plots (Figure 3B). MV-generating apoptotic splenocytes were in a comparable phase of apoptosis (Figure 3C) and the percentage of apoptotic MVs originating from splenocytic T or B cells was comparable for MRL/lpr and BALB/c (Figure 3D). In summary, apoptotic MVs generated *in vitro* from splenocytes of MRL/lpr mice expose more (modified) chromatin compared to BALB/c splenocytes.

### Splenocyte-derived apoptotic microvesicles from MRL/lpr mice are more potent activators of DC than microvesicles from BALB/c mice

**Figure 4:**
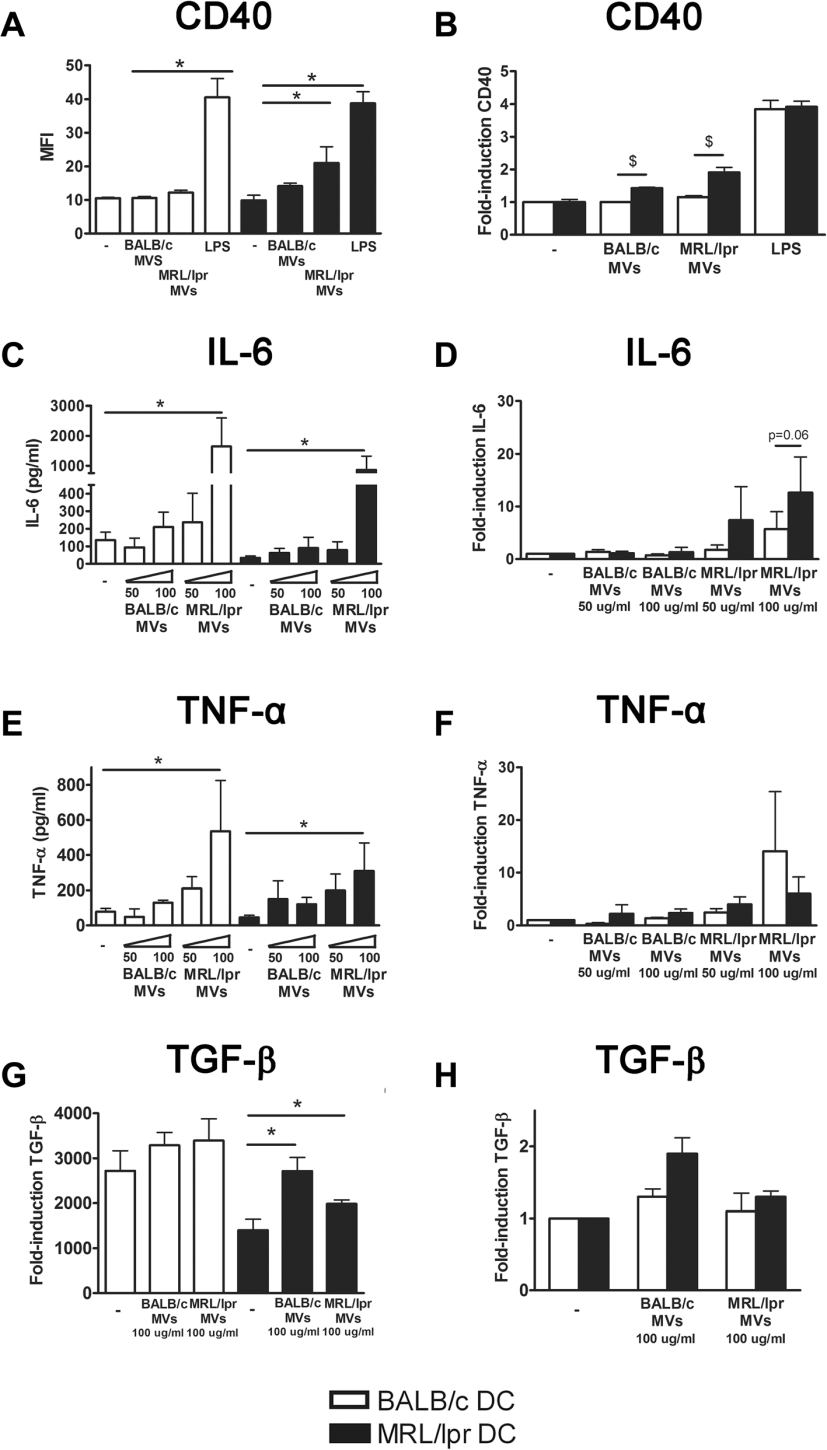
Splenocyte-derived apoptotic microvesicles from MRL/lpr mice, and not those from BALB/c mice, are able to activate MRL/lpr dendritic cells (DC), that are also more prone to activation compared to BALB/c DC. CD40 expression **(A)**, and IL-6 **(C)**, TNF-α **(E)** and transforming growth factor (TGF)-β **(G)** levels produced by BALB/c and MRL/lpr DC were measured after stimulation with the indicated concentration of splenocyte-derived apoptotic microvesicles. The relative CD40 expression **(B)**, and IL-6 **(D)**, TNF-α **(F)** and TGF-β **(H)** levels of microvesicle- or LPS-stimulated MRL/lpr DC were compared to BALB/c DC.*P <0.05 compared to unstimulated DC, ^$^ *P* <0.05 compared to DC from BALB/c. MFI, mean fluorescence intensity.

In MRL/lpr DC, MRL/lpr splenocyte-derived MVs induced a significantly higher expression of CD40 than BALB/c splenocyte-derived MVs (Figure 4A). Splenocyte-derived MVs of both BALB/c and MRL/lpr mice induced a significantly higher expression of CD40 in MRL/lpr DC, compared to BALB/c DC (Figure 4B). MRL/lpr splenocyte-derived MVs induced production of IL-6 and TNF-α in both MRL/lpr and BALB/c DC, while BALB/c MVs had no significant effect (Figure 4C and E). The MV-induced IL-6 production tended to be more pronounced in MRL/lpr DC (*P* = 0.06) (Figure 4D), but this was not the case for TNF-α (*P* = 0.84) (Figure 4F). Both MRL/lpr and BALB/c MVs significantly induced TGF-β production in MRL/lpr DC, but not in BALB/c DC (Figure 4G and H). There was correlation between the MV-induced production of IL-6 by DC and the MFI of the respective MV-samples for mAbs BT164 (*r*^2^ = 0.53, *P* = 0.02) and LG11-2 (*r*^2^ = 0.49, *P* = 0.03). Taken together, MVs derived from MRL/lpr splenocytes, and not those from BALB/c splenocytes, were able to activate DC. The potency to induce the pro-inflammatory cytokine IL-6 appeared to be correlated with the amount of exposed apoptosis-modified chromatin in the MVs. As described above for 32Dcl3-derived MVs, MRL/lpr DC also appeared relatively more sensitive to activation by splenocyte-derived MVs.

### Plasma-derived microvesicles from MRL/lpr mice expose more chromatin than microvesicles in plasma from BALB/c mice

**Figure 5:**
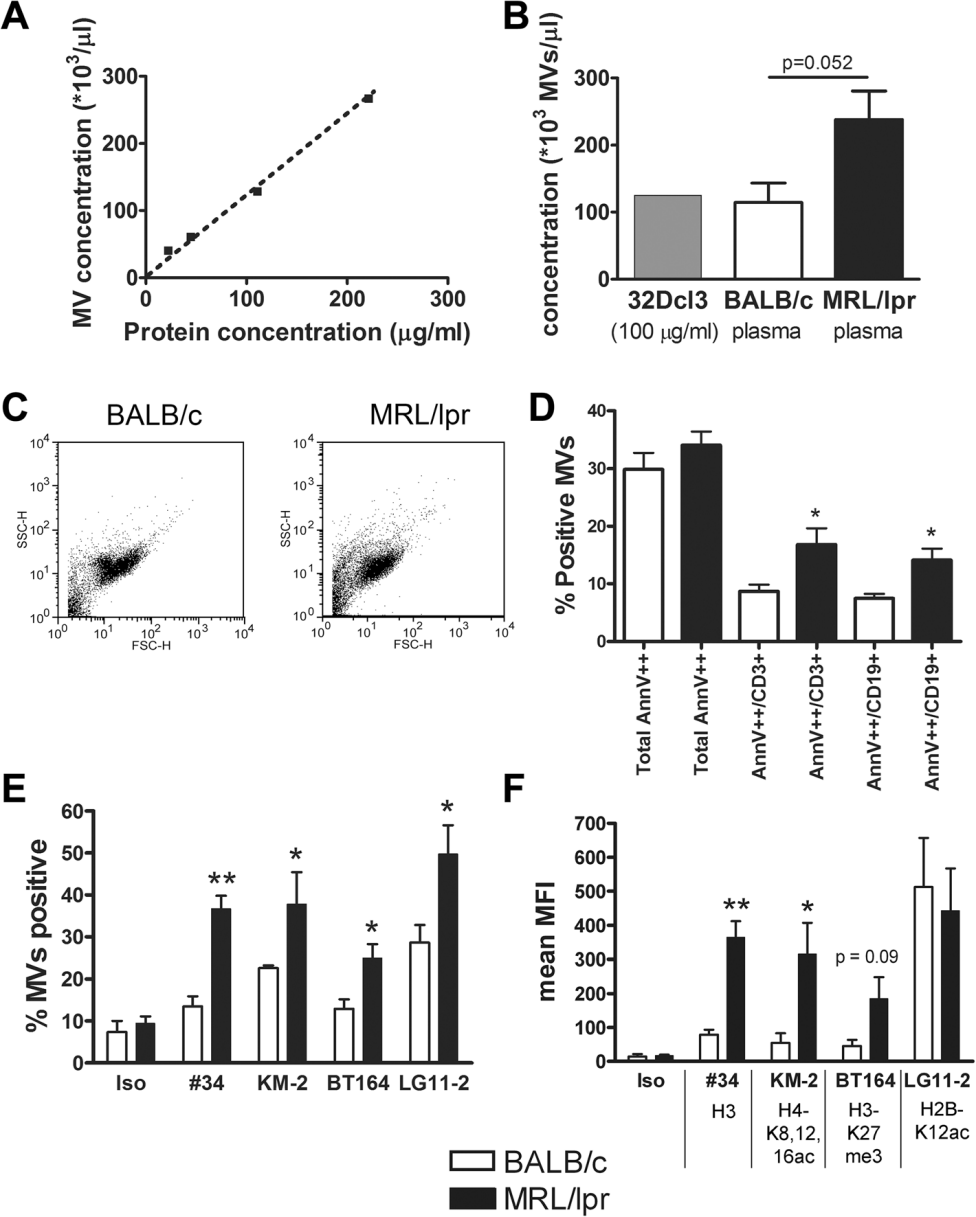
Plasma-derived microvesicles (MVs) from MRL/lpr mice expose a higher amount of chromatin and contain a higher percentage of annexin V^++^/CD3^+^ and annexin V^++^/CD19^+^ MVs. **(A)** The concentration of apoptotic MVs derived from 32Dcl3 cells determined in flow cytometry using counting beads (y-axis) was compared to the protein concentration of the same MV samples measured by the BCA assay (x-axis). **(B)** The concentration of MVs isolated from apoptotic 32Dcl3 cells, brought to a concentration of 100 μg protein/ml, and from plasma of 8- to 10-week-old BALB/c and MRL/lpr mice were determined by flow cytometry. **(C)** Representative forward scatter/side scatter (FSC/SSC) plots obtained from plasma-derived MVs of a BALB/c and MRL/lpr mouse to demonstrate comparable MV populations. **(D)** The percentage of MVs positive for Annexin V, anti-CD3-PE or anti-CD19-PE, was measured by flow cytometry for MRL/lpr mice in comparison to BALB/c mice (both n = 8). **(E,** **F)** Flow cytometry of plasma-derived MVs from BALB/c and MRL/lpr mice after staining with the indicated mAbs. The percentage positive MVs **(E)** and the mean fluorescence intensity (MFI) **(F)** of MRL/lpr MVs was compared to BALB/c microvesicles. *P <0.05; **P <0.01.

The concentration of MVs from plasma of MRL/lpr and BALB/c mice analyzed by flow cytometry revealed a linear correlation with the protein concentration using 32Dcl3-derived MVs as a standard (Figure 5A). A protein equivalent of 100 μg/ml of 32Dcl3-derived MVs corresponded to approximately 10^5^ MVs/μl, which was comparable to the mean concentration of MVs in plasma of BALB/c mice, while MRL/lpr mice tended to have higher concentrations (p = 0.052) (Figure 5B). Plasma-derived MVs did not show large differences in the FSC/SSC plot between both mice strains (Figure 5C). Although the total Annexin V^++^ population showed no proportional difference, MRL/lpr MVs contained a significantly higher percentage of Annexin V^++^/CD3^+^ (T cell-derived) and Annexin V^++^/CD19^+^ (B cell-derived) MVs compared to BALB/c MPs (Figure 5D). Annexin V^++^/CD3^+^ and Annexin V^++^/CD19^+^ MVs formed together approximately 90% of the total population of Annexin V^++^ MVs. Importantly, MRL/lpr plasma also contained a higher percentage of MVs positive for our panel of anti-chromatin mAbs (Figure 5E). The mean fluorescent intensity/MV for mAb #34 and KM-2 was significantly higher for MRL/lpr MVs, while BT164 also tended to be higher (Figure 5F). For LG11-2, we observed a high mean fluorescent intensity, but a lower percentage of LG11-2-positive MVs in BALB/c mice, which was due to a small (unidentified) subpopulation of MVs. In summary, plasma from MRL/lpr mice contain an increased percentage of CD3^+^/CD19^+^ MVs with an increased amount of (modified) chromatin compared with MVs in plasma of BALB/c mice.

### Plasma-derived microvesicles of MRL/lpr mice are more potent activators of DC and splenocytes than microvesicles from BALB/c mice

**Figure 6:**
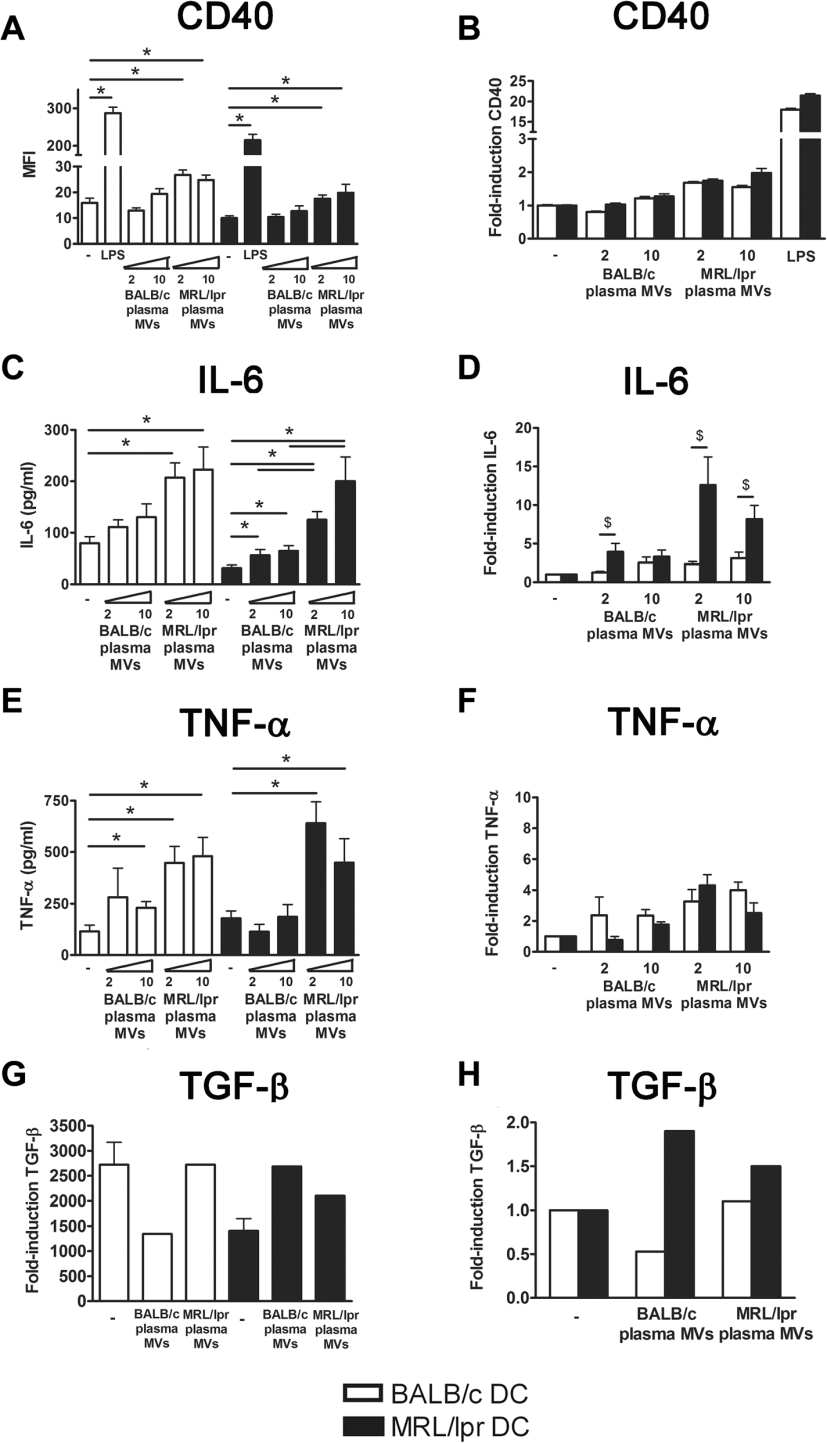
Microvesicles (MVs) isolated from plasma of MRL/lpr mice are more potent in activating dendritic cells (DC) compared to BALB/c microvesicles. CD40 expression **(A)** and IL-6 **(C)** TNF-α **(E)** and transforming growth factor (TGF)-β **(G)** levels produced by BALB/c and MRL/lpr DC were measured after stimulation with the indicated MVs. The relative CD40 expression **(B)** and IL-6 **(D)** TNF-α **(F)** and TGF-β **(H)** levels of MV- or lipopolysaccharide (LPS)-stimulated MRL/lpr DC were compared to BALB/c DC. *P <0.05 compared to unstimulated DC or BALB/c plasma-derived microvesicles, ^$^ *P* <0.05 compared to BALB/c DC.

**Figure 7:**
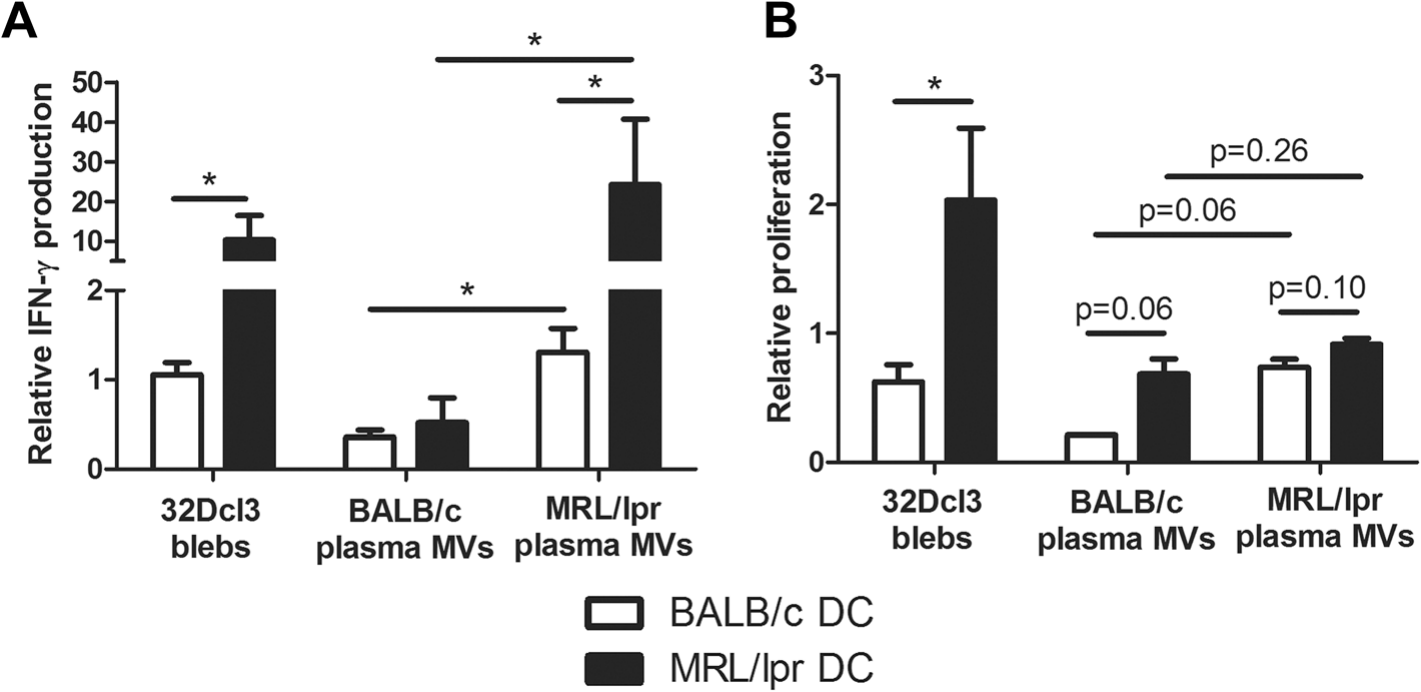
Dendritic cell (DC)-mediated splenocyte activation is increased in MRL/lpr DC stimulated with plasma-derived microvesicles from MRL/lpr mice. **(A)** Relative IFN-γ production of CBA splenocytes was measured after incubation with microvesicle (MV)-stimulated DC and compared to splenocytes incubated with unstimulated DC. **(B)** Relative proliferation of carboxyfluorescein succinimidyl ester (CFSE)-labeled CBA splenocytes was determined in mixed lymphocyte reaction after incubation with MV-stimulated DC and compared to splenocytes incubated with unstimulated DC. *P <0.05.

Only MVs from MRL/lpr mice were able to induce a significant CD40 expression in both BALB/c and MRL/lpr DC (Figure 6A). MV-induced CD40 expression was not significantly higher in MRL/lpr DC, however, a trend could be seen for the highest concentration of MRL/lpr MVs (Figure 6B). Plasma-derived MVs from MRL/lpr mice also induced the production of IL-6 and TNF-α, while MVs from BALB/c mice induced only a small increase (Figure 6C and E). Calculation of the ratio of MV-induced cytokine production compared to unstimulated DC confirmed that the MV-induced relative increase in IL-6 production was considerably higher for MRL/lpr DC compared to BALB/c DC (Figure 6D). The MV-induced relative increase in TNF-α did not differ between MRL/lpr and BALB/c DC (Figure 6F). Plasma of 13- and 18-weeks-old BALB/c and MRL/lpr mice essentially gave similar results (data not shown). In addition, MVs from both MRL/lpr and BALB/c mice induced TGF-β in MRL/lpr DC (Figure 6G and H). Finally, we compared the splenocyte-activating capacity of DC incubated with 32Dcl3- or plasma-derived MVs (Figure 7). MRL/lpr DC incubated with 32Dcl3- or plasma-derived MVs from MRL/lpr mice induced a 10-20-fold increase in IFN-γ production by MHC-mismatched CBA splenocytes (Figure 7A). BALB/c DC and/or BALB/c MVs reduced or induced only a small increase in the production of IFN-γ. Similar trends could be observed for the proliferation of splenocytes after incubation with MV-stimulated DC, although these differences were rather small for DC stimulated with plasma-derived MVs (Figure 7B). In summary, plasma-derived MVs from MRL/lpr mice are more potent stimulators of DC activation, in particular it seems of MRL/lpr DC, compared to MVs derived from BALB/c mice.

## Discussion

We observed that both *in vitro*-generated and *ex vivo*-isolated MVs from MRL/lpr mice contained an increased amount of accessible (modified) chromatin compared to MVs from BALB/c mice. Chromatin in MVs of MRL/lpr mice might posses a more open structure due to an increased acetylation of histones H2A/H4 and H2B, which we observed here. For the *in vitro*-generated MVs, we could exclude differences in rate/stage of apoptosis, in composition of splenocytes (data not shown), and in a subset of splenocytes going into apoptosis, as sources for the increased amount of chromatin. Apoptotic MVs from both MRL/lpr and BALB/c mice still contained intact nucleosomes and dsDNA (data not shown), which excludes a large-scale breakdown of nucleosomes as a source of increased accessibility. Dysfunctional apoptotic processing could lead to other structural differences of chromatin in MVs of lupus mice; for example, a reduced fragmentation of apoptotic chromatin has been demonstrated in kidneys of NZBW/F1 lupus mice [32]. However, future research has to determine the exact cause for the increased amount or accessibility of (modified) chromatin in apoptotic MVs from MRL/lpr mice.

In plasma from both 8- to 10-week-old BALB/c and MRL/lpr mice there is a similar number of MVs that are highly positive for Annexin-V. Another study also observed that BALB/c and lupus mice contained comparable amounts of MVs [23]. In addition, we have previously shown that apoptotic nucleosomes can be detected both in the circulation of BALB/c and MRL/lpr mice at a young age [8]. MRL/mpj mice have a similar genetic background to MRL/lpr, and are often used as control mice. However, MRL/mpj mice have defective removal of apoptotic cells that is similar to MRL/lpr mice [33,34]. In addition, as with MRL/lpr mice, MRL/mpj mice have persistent high levels of apoptotic nucleosomes in their circulation at a later age [8]. Therefore, we chose to use BALB/c mice as control mice for this study. As the concentration of MVs in MRL/lpr mice and BALB/c mice is similar, the immunostimulatory capacity of circulating (apoptotic) MVs in murine SLE could be explained by differences in composition. Indeed, we show that the proportion of circulating apoptotic MVs that is derived from B and T cells, is significantly higher for MRL/lpr mice compared to BALB/c mice. This may be explained by an increased number of lymphocytes that escape selection in the thymus and enter the circulation of MRL/lpr mice [35,36]. Moreover, the vast majority of circulating apoptotic MVs appeared to be derived from T or B cells, whereas we did not detect a significant amount of endothelial-derived MVs (not shown). To our knowledge, increases in B or T cell-derived apoptotic MVs have not been described in lupus mice or SLE patients. In addition, CD3- or CD19-positive MVs are elevated in patients with other autoimmune diseases [16,37] and patients with chronic lymphoproliferative diseases [38].

We have previously shown that apoptotic MVs from 32Dcl3 cells are capable of maturing DC from normal mice, leading to a mixed Th1/Th17 response [12,13]. We now show that *in vitro*-generated MVs and *ex vivo*-isolated MVs derived from MRL/lpr mice were more potent activators of DC than MVs and MVs from BALB/c mice. In fact, MVs derived from BALB/c mice, showed virtually no activation of DC. In addition, DC-mediated activation of splenocytes was also increased after incubation with MRL/lpr MVs. Importantly, MVs were isolated from 8- to 10-week-old pre-diseased MRL/lpr mice that did not possess detectable amounts of anti-DNA autoantibodies in their circulation (data not shown), to exclude a contribution of immune complexes in the activation of DC. In addition, we found no MV-bound IgG in 8- to 10-week-old MRL/lpr mice, using a labeled anti-mouse IgG antibody (data not shown), which has been described for MRL/lpr mice >14 weeks old [23]. The observed correlation between the amount of exposed chromatin in splenocyte-derived MVs and their potency to induce IL-6 suggests that the increased exposure of apoptosis-modified chromatin on MRL/lpr MVs might be related to their increased immunogenicity. Indeed, histones are capable of activating the innate immune system [39,40]. Moreover, we have previously shown that acetylation of histones and chromatin increases their immunogenicity *in vitro* and *in vivo* [25]. Interestingly, Fehr *et al*. have recently demonstrated a different modulating effect of apoptotic MVs, which were *ex vivo*-generated from human lymphocytes, on DC from healthy individuals and SLE patients [41].

Our finding that MRL/lpr DC produced lower basal and LPS-induced levels of IL-6 is in accordance with a previous study showing that DC from lupus mice, including MRL/lpr and MRL/mpj, produce lower levels of IL-6 in response to several toll-like receptor (TLR)-ligands [42]. This was attributed to a diminished ability to sustain IκBα phosphorylation, resulting in lower levels of IL-6 mRNA. Lower production of IL-6 in response to CpG has also been found in peripheral blood mononuclear cells from SLE patients [43]. In contrast to the lower basal levels, the fold-induction of IL-6 by *in vitro*-generated and *ex vivo*-isolated apoptotic MVs was relatively higher in MRL/lpr DC compared to BALB/c DC. Notably, IL-6 plays a dual role because it is involved both in immunity and tolerance. IL-6 is essential for the induction of a Th17 response and suppression of Treg [44], but also regulates the maturation of DC [45,46]. IL-6 knockout mice show increased numbers of matured DC [45]. Therefore, lower basal IL-6 production by DC might affect their differentiation/maturation and render them more susceptible to certain stimuli, whereas the subsequently enhanced IL-6 production might promote a Th17 response. Our findings indicate that TGF-β, a cytokine that is also able to inhibit DC maturation and which cooperates with IL-6 to induce a Th17 response, behaved quite similarly to IL-6 in MRL/lpr DC. In contrast, the MV-induced production of TNF-α was not enhanced in MRL/lpr DC. This may be explained by the fact that the pathways used to induce IL-6 and TNF can differ [47].

## Conclusions

We showed that in the MRL/lpr lupus mouse model, apoptotic MVs are more potent activators of DC, which was associated with an increased content of apoptosis-modified chromatin. Our data suggest that the composition of the chromatin in MVs dictates their immunogenicity. Breaking the tolerance in MRL/lpr mice might also be facilitated by a relative enhanced sensitivity of their DC for these immunogenic apoptotic MVs. Future studies into the exact properties of the immunogenic apoptotic MVs and their receptors on DC, in particular also in SLE patients, should lead to a better insight into the processes that result in breaking of tolerance in SLE.

## Abbreviations

BSA: bovine serum albumin
CFSE: carboxyfluorescein succinimidyl ester
DC: dendritic cells
ELISA: enzyme-linked immunosorbent assay
FCS: fetal calf serum
FITC: fluorescein isothiocyanate
FSC: forward scatter
IFN: interferon
IL: interleukin
LPS: lipopolysaccharide
mAb: monoclonal antibody
MFI: mean fluorescence intensity
MLR: mixed lymphocyte reaction
MV: microvesicle
PBA: phosphate buffered saline with 0.1% bovine serum albumin
PBS: phosphate-buffered saline
PI: propidium iodide
RPMI: Roswell Park Memorial Institute medium
SLE: systemic lupus erythematosus
SSC: side scatter
TGF: transforming growth factor
TNF: tumor necrosis factor
